# The Curious Case of Multicellularity in the Volvocine Algae

**DOI:** 10.3389/fgene.2022.787665

**Published:** 2022-02-15

**Authors:** Berenice Jiménez-Marín, Bradley J. S. C. Olson

**Affiliations:** ^1^ Division of Biology, Kansas State University, Manhattan, KS, United States; ^2^ Interdepartmental Genetics Graduate Program, Kansas State University, Manhattan, KS, United States

**Keywords:** multicellularity, green algae, volvocine algae, co-option, gene loss, developmental complexity, multicellular evolution

## Abstract

The evolution of multicellularity is a major evolutionary transition that underlies the radiation of many species in all domains of life, especially in eukaryotes. The volvocine green algae are an unconventional model system that holds great promise in the field given its genetic tractability, late transition to multicellularity, and phenotypic diversity. Multiple efforts at linking multicellularity-related developmental landmarks to key molecular changes, especially at the genome level, have provided key insights into the molecular innovations or lack thereof that underlie multicellularity. Twelve developmental changes have been proposed to explain the evolution of complex differentiated multicellularity in the volvocine algae. Co-option of key genes, such as cell cycle and developmental regulators has been observed, but with few exceptions, known co-option events do not seem to coincide with most developmental features observed in multicellular volvocines. The apparent lack of “master multicellularity genes” combined with no apparent correlation between gene gains for developmental processes suggest the possibility that many multicellular traits might be the product gene-regulatory and functional innovations; in other words, multicellularity can arise from shared genomic repertoires that undergo regulatory and functional overhauls.

## Introduction

The evolution of multicellular organisms is a major Transition in Evolution where unicells relinquish their individuality to collectively coordinate for the development of a complex, higher organizational level individual (John Maynard Smith, 1995; Grosberg and Strathmann, 2007; Knoll, 2011). Multicellularity appears to be a successful solution for adapting to the environment; it evolved independently multiple times in all domains of life (Bonner, 2000; Grosberg and Strathmann, 2007; Ruiz-Trillo and Nedelcu, 2015) having generated extant descendants that showcase a wealth of morphological and developmental innovation. Unicellular organisms have been shown to readily evolve multicellular lifestyles in response to selection (Grosberg and Strathmann, 2007) suggesting that the genetic requirements for multicellularity are not a major hurdle for the transition itself to occur (Rokas, 2008; Arenas-Mena, 2017).

Indeed, experimental evolution studies demonstrate the rapid ability of unicells such as yeast (Ratcliff et al., 2013), and green algae (Boraas et al., 1998; Sathe and Durand, 2015; Fisher et al., 2016; Herron et al., 2019) to transition to multicellularity. Although the recurring nature of the evolution of multicellularity provides a useful base for comparative studies, determining the mechanisms underlying the evolution of multicellularity remains enigmatic due to technical obstacles such as long divergence times between multicellular models and their unicellular relatives and limited toolkits for experimental work in multicellularity models. Tractable models for understanding the evolution of multicellularity are thus indispensable to define the molecular players underlying multicellular evolution. A curious clade of chlorophytes, the volvocine algae, are an important system for addressing the molecular basis of multicellular evolution.

**FIGURE 1:**
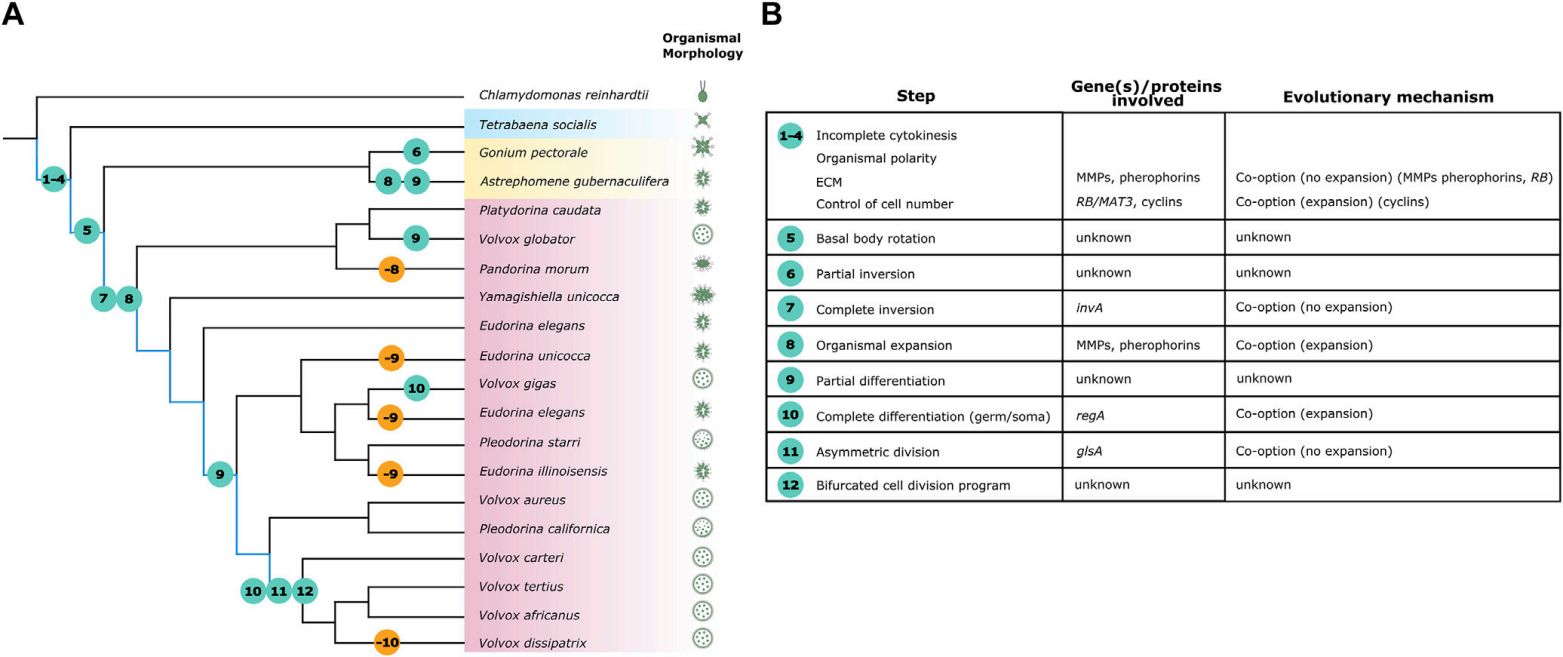
The evolution of volvocine developmental complexity is marked by gains and losses of traits. Adapted from (Ruiz-Trillo and Nedelcu, 2015; Lindsey et al., 2021). **(A)** Phylogeny of the volvocine algae. Multicellular volvocine families Tetrabaenaceae, Goniaceae, and Volvocaceae are demarcated in blue, yellow, and pink respectively. Kirk’s lineage explanation for volvocine multicellularity is highlighted in blue. Traits associated to Kirk’s twelve steps are marked by circles; numbers within circles detail what step is described. Teal circles represent evolution of traits and orange circles represent loss of traits. **(B)** Organismal morphology for depicted species is not to scale. **(B)** Kirk’s twelve steps do not always have a known genetic origin, and when they do, it is not always co-opted *via* duplication and divergence.

The volvocines are a well-established model system for the study of multicellularity (Nedelcu and Michod, 2003; Kirk, 2005; Miller and Technau, 2010; Niklas, 2014; Grochau-Wright et al., 2017) that encompasses ∼50 extant multicellular species and their closest unicellular relative, *Chlamydomonas reinhardtii* (Kirk, 1997; Coleman, 1999; Umen and Olson, 2012) (Figure 1A). Multicellular volvocines are arranged into families Tetrabaenaceae (*Basichlamys, Tetrabaena*), Goniaceae (*Gonium, Astrephomene*) and Volvocaceae (*Pandorina, Eudorina, Yamagishiella, Volvox*) (Hanschen et al., 2018a; Lindsey et al., 2021), and display morphologies ranging from bowl-shaped undifferentiated colonies to differentiated spheroids (Kirk, 1997; Coleman, 2012; Yamashita et al., 2016) (Figure 1A). Despite their phenotypic differences, this diverse group of algae evolved from their *Chlamydomonas-*like unicellular ancestor relatively recently during the Triassic period (around 250 MYA) (Herron et al., 2009). The late evolution of multicellularity of the volvocines has made them attractive subjects for comparative genomic analyses, which have shown that their genomes are highly similar and share conserved synteny (Prochnik et al., 2010; Hanschen et al., 2016; Featherston et al., 2017; Jiménez-Marín et al., 2021). The remarkable genetic similarity between volvocine species suggests the genetic signals relating to multicellularity might still be traceable within the lineage and makes this algal system suitable for the study of multicellularity at the molecular level. The genetic tractability of the volvocines, coupled to the increasing availability of functional genomic tools in key species (Lerche and Hallmann, 2009; Umen and Olson, 2012; Lerche and Hallmann, 2013), their phenotypic diversity and recent divergence from a *Chlamydomonas*-like ancestor*,* provide a unique framework for determining the common principles and functional players behind multicellularity, which impacts the broader fields of evolution and development.

### Variations on a Theme—Evolution of Development in the Volvocine Algae

The seeming stepwise acquisition of phenotypes that the volvocines display (from unicellular to undifferentiated and differentiated multicellular) have been used as the basis for a hypothesis of sequential acquisition of developmental complexity through genetic co-option (Kirk, 2005; Olson and Nedelcu, 2016). David Kirk’s seminal work on this subject suggested that twelve developmental changes could explain the evolution of differentiated multicellularity in *Volvox carteri* (Kirk, 2005) (Figure 1A, highlighted in blue). At least four of Kirk’s developmental changes are required for undifferentiated multicellularity as exhibited by *Tetrabaena* and *Basichlamys* (Figure 1)*.* The best described undifferentiated multicellular species, *Gonium pectorale*, covers six of Kirk’s twelve steps (Umen and Olson, 2012) (Figure 1), suggesting that within the volvocines, the evolution of undifferentiated multicellularity requires the most developmental changes.

The lineage hypothesis of co-option proposes that volvocine multicellularity resulted from modification of cellular structures and developmental cycles under an otherwise conserved phenotypic background (Kirk, 1997; Bonner, 2000). This appears to be true for volvocine morphology; except for species with differentiated germ cells, the cells that compose volvocine colonies strongly resemble their closest unicellular relative, *Chlamydomonas. Chlamydomonas* cells are ovoid shaped and asymmetrical (Holmes and Dutcher, 1989) (Figure 1A). At their anterior end, each cell has two flagella that serve chemosensory and motility functions. At their posterior end, cells have a distinctive large cup-shaped chloroplast that occupies the bulk of the intracellular space (Umen and Olson, 2012). Multicellular volvocine cells are enveloped in a proteinaceous extracellular matrix (ECM) that resembles the ECM of animal cells rather than cellulosic cell walls of plants (Sumper and Hallmann, 1998). Interestingly, the multicellular volvocine ECM is biochemically and genetically conserved with *Chlamydomonas’* cell wall (Adair et al., 1987; Prochnik et al., 2010; Hanschen et al., 2016), suggesting the ancestral genes were repurposed for new multicellularity functions. Different species secrete various amounts of ECM, with the most extreme example being *Volvox.* Cells in *Volvox* are enveloped in ECM layers such that it comprises roughly 99% of the spheroid volume (Kirk, 1997).

Likewise, the multicellular volvocine life cycle is very similar to that of *Chlamydomonas*. All volvocines are haploid and capable of both vegetative and sexual reproduction (Hoops et al., 2006). Vegetative reproduction occurs in individual cells that undergo a modified cell cycle called “multiple fission” (Cross and Umen, 2015). Except for germ cells in *Volvox*, every volvocine cell is flagellated and hence provides motility to the whole organism until cell division occurs, at which point the cells retract their flagella, undergo division, daughter cells regrow their flagella and begin swimming again. Given that the volvocine cells are not symmetrical, in multicellular species the organism as a whole must retain defined cell orientations in the context of their bowl (e.g., *Gonium*) or spheroid (e.g., *Pandorina, Eudorina, Pleodorina* and *Volvox*) morphologies and the number of cells in the colony for the organism to successfully swim (Hoops, 1993).

At the molecular level, one of key genetic innovations for volvocine multicellularity is co-option of the cell cycle regulator and tumor suppressor ortholog Retinoblastoma (RB). RB (encoded by the *MAT3* gene) from multicellular *Gonium* causes unicellular *Chlamydomonas* to be multicellular (Hanschen et al., 2016). As in plants and animals, all volvocines have an RB ortholog that regulates their cell cycles through cyclin-dependent kinase (CDK) phosphorylation (Ferris et al., 2010; Hanschen et al., 2016). RB from multicellular volvocine species differ in their regulatory domains from their ortholog in unicellular *Chlamydomonas*. First, RB from multicellular volvocines have shorter linkers between the RB-A and RB-B domains compared to *Chlamydomonas* (Ferris et al., 2010; Hiraide et al., 2013; Hanschen et al., 2016; Featherston et al., 2017). Second, RB from multicellular volvocines lack conservation in C-terminal domain and CDK phosphorylation sites that are present in their unicellular relative (Hiraide et al., 2013; Hanschen et al., 2016; Featherston et al., 2017). Additionally, *Volvox carteri* has different sex-based isoforms of RB that correlate with oogamy (Ferris et al., 2010). While cases of gene family expansion in the volvocines are limited, several multicellular volvocines, including *Gonium*, show expansion of cyclin D1 gene family (Prochnik et al., 2010; Hanschen et al., 2016). Cyclin D genes regulate the cell cycle by dimerizing with CDKs and phosphorylating RB proteins (Cross and Umen, 2015; Hanschen et al., 2016; Featherston et al., 2017; Jiménez-Marín et al., 2021); whether cyclins are involved in the evolution of volvocine multicellularity is yet to be determined. Regardless of the impact of cyclins, it would seem likely that cell cycle dependent regulation of gene expression by RB is different for *Chlamydomonas* compared to its multicellular volvocine relatives, possibly driving the evolution of multicellular development in the latter.

Aside from RB and cyclin D1s, other known co-option events do not seem to coincide with Kirk’s six steps to undifferentiated multicellularity (Figure 1B, steps 1–6). For instance, “ECM-related” genes, which provide structure and cohesion to volvocine colonies, were likely co-opted for multicellularity. However, *Gonium* and *Chlamydomonas* share similar numbers of key ECM-related genes despite the former being multicellular and the latter unicellular (Hanschen et al., 2016; Olson and Nedelcu, 2016; Jiménez-Marín et al., 2021). Instead, expansion and diversification of ECM-related genes (pherophorins, matrix metalloproteinases- MMPs) seems to relate more to changes in organismal size than to the evolution of colonial life (Prochnik et al., 2010; Hanschen et al., 2016; Featherston et al., 2017; Jiménez-Marín et al., 2021). Similarly, genes that became co-opted for key volvocine developmental processes originated much before the appearance of the phenotypes with which they are associated (Figure 1).

Differentiated multicellularity in the volvocines has been long thought to be a consequence of developmental innovations that originate from further modification of structures and co-option of genes present in undifferentiated multicellular ancestors, and likely in extant undifferentiated sister species. Spheroidal and bowl shaped volvocine embryos need to undergo a process akin to gastrulation in which the colony shape must change to ensure a final configuration that allows the organism to swim (Hallmann, 2006). This process, called inversion, is known to occur in the Goniaceae [all but *Astrephomene* (Yamashita et al., 2016)], and Volvocaceae (Hallmann, 2006). The product of *invA*, a kinesin, is known to play a key role in inversion in *Volvox.* Orthologs of *invA* are present in several volvocine species, regardless of their morphology, and noteworthily, *Chlamydomonas* also has an ortholog for this gene, *iar1*. Surprisingly, the *Chlamydomonas iar1* can rescue *Volvox* inversion-defective *invA* mutants (Nishii et al., 2003). However, whether *Gonium’s* ortholog of *invA* has a role in inversion remains to be demonstrated. Likewise, co-chaperone *glsA*, which is involved in asymmetric cell division in *Volvox*—a process tied to cell differentiation in this and other volvocines, has orthologs in species that do not undergo cell differentiation (Jiménez-Marín et al., 2021). The *Chlamydomonas* ortholog for *glsA*, much like *iar1*, can rescue gonidialess *glsA Volvox* mutants (Cheng et al., 2003).

Perhaps the best-known example of co-option for a key volvocine developmental processes preceding the phenotype itself is *Volvox*’s cell differentiation master regulator regA. Whilst there are *regA* homologs in *Chlamydomonas* and *Gonium* (*RLS1*), there are no orthologs of it in either species (Hanschen et al., 2014; Hanschen et al., 2016). In the species that have it, *regA* is part of a gene cluster that likely evolved through tandem duplication shortly after Goniaceae split from Volvocaceae (Hanschen et al., 2014; Grochau-Wright et al., 2017). Indeed, the *Pandorina* (Volvocaceae) genome encodes for the full *reg* cluster, despite *Pandorina* being an undifferentiated species (Grochau-Wright et al., 2017). Ancestral state reconstruction of the *reg* cluster supports the hypothesis that the genetic basis for somatic cell differentiation in the Volvocaceae preceded cellular differentiation itself and was subject to co-option (Grochau-Wright et al., 2017). A recent transcriptome study of *Volvox* gonidia and somatic cells demonstrated the different cell types have markedly distinct transcriptional programs, and suggest that co-option of certain gene expression programs for differentiation might have driven cell type specialization (Matt and Umen, 2018). A major open question in the field is whether regA acts as a key regulator of these transcriptional programs during development to establish the germ and somatic cell lineages (Olson and Nedelcu, 2016).

Traits related to sexual reproduction are oftentimes highly dynamic evolutionarily. In the case of the volvocines, there are members whose reproductive morphology range from isogamous (*Chlamydomonas, Gonium, Pandorina*) to anisogamous (*Eudorina*) and oogamous (*Volvox*). However, the evolution of anisogamy does not appear to be correlated to the evolution of cellular differentiation, though it does correlate with increased complexity (Hanschen et al., 2018a). The mating-type (MT) loci of different volvocines experience frequent turnover, and their distribution (as well as that of MT linked genes) does not seem to relate to the evolution of anisogamy within the clade. Thus, it is hypothesized that the evolution of volvocine males might be the consequence of altering the function of *MID*, a sex determining putative transcription factor, or of its target genes, as opposed to the acquisition of novel genes controlling gamete size (Hamaji et al., 2018). Empirical, phylogenetics-based evidence supports predictions that multicellularity is a driver of derived sexual traits, starting with anisogamy and then with sexual dimorphism (Hanschen et al., 2018a).

While the co-option of *RB, glsA, regA* and other examples here given demonstrates that shared genetic elements can be repurposed for a wide array of biological roles, surprisingly few ‘multicellularity genes’ are found in the volvocines (Figure 1B). Unlike the initial shift to undifferentiated multicellularity that was likely boosted by co-option of *RB*, it would seem like subsequent genetic changes related to ECM maintenance, inversion, and cell differentiation have roles in stabilizing colonial life and controlling subsequent developmental processes rather than impacting multicellularity itself (Figure 1B). Indeed, Kirk’s streamlined framework has undergone revisions in light of the discovery of a dynamic history of gains and losses of morphological and developmental traits (Nanjundiah et al., 2018; Lindsey et al., 2021) (Figure 1A). Hence, the volvocine phylogenetic tree is under constant revision (Nozaki et al., 2003; Hanschen et al., 2018b; Lindsey et al., 2021). Genera *Eudorina, Pleodorina* and *Volvox* are not monophyletic (Figure 1A), and family Goniaceae likely is not either (Lindsey et al., 2021). Ancestral state reconstruction shows that there have been repeated instances of evolution of cell differentiation and loss of cell differentiation within the volvocines (Grochau-Wright et al., 2017; Lindsey et al., 2021) (Figure 1A). Other landmark traits, including the evolution of spheroidal morphologies, inversion, and expansion/contraction of the ECM, seem to have occurred independently more than once as well (Herron et al., 2009; Ruiz-Trillo and Nedelcu, 2015) (Figure 1A). The most extreme example of independent evolution is *Astrephomene*, a proposed (but debated) sister genus of *Gonium*, which evolved a spheroidal shape, has sterile somatic cells without the *reg* cluster, and rearranges itself without undergoing inversion (Yamashita et al., 2016). Upon inspecting gains and losses in morphology, it would seem that the transition to undifferentiated multicellularity is not as readily gained as lost, again supporting the idea that this step is more developmentally complex to evolve. The frequent shifts between simplification and complexification of traits along multicellular volvocine history paint a picture that does not support widespread genetic innovation as a driver of developmental complexity.

### How can Developmental Complexity Arise From a Widely Shared Genomic Repertoire?

One of the most striking features of multicellular evolution in the volvocine algae is how few new genes seem to be required to drive complex developmental changes. Moreover, many such genes are largely conserved in 1:1 orthology across the entire lineage (e.g., *invA*, *glsA*, RB/MAT3) and relatively few examples of gene expansion (e.g., cyclin Ds, *regA,* MMPs). Surprisingly, comparative analyses of multicellular volvocine genomes to unicellular *Chlamydomonas* (Jiménez-Marín et al., 2021) combined with experimental approaches support the impression that few key genes were co-opted for volvocine developmental traits, and that other processes such as *de novo* gene evolution are much less important (Hanschen et al., 2016; Olson and Nedelcu, 2016; Jiménez-Marín et al., 2021) to multicellularity in this system. Thus, alternative mechanisms must be behind the dynamic developmental and evolutionary picture the volvocine algae have painted.

Inferences on the minimal role of genetic innovation made through phylogenetic approaches have been confirmed through comparative genomics. Orthologous groups, protein family (Pfam) domains, regulatory protein families, and even histones except for H1 are less numerous in *Volvox* than they are in less developmentally complex volvocines. Contraction of shared groups among the genomes of *Gonium, Pandorina, Yamagishiella, Eudorina* and *Volvox* relative to *Chlamydomonas* is more frequent than is expansion of other shared elements (Jiménez-Marín et al., 2021). Interestingly, the highly conserved synteny between volvocine genomes facilitates mechanistic analysis of gene loss (that is, analysis of what genes have been lost and the manner of their loss). This analysis shows that losses mainly occur through progressive decay (Jiménez-Marín et al., 2021). What is more, a significant proportion of analyzed gene losses are common to *Gonium, Pandorina, Yamagishiella, Eudorina* and *Volvox* but not to *Chlamydomonas*, strongly suggesting that bursts of gene loss at the last common ancestor (LCA) of the multicellular volvocines might have played a significant role in the transition to multicellularity of this lineage (Jiménez-Marín et al., 2021).

The high sequence similarity shared among volvocine genomes has allowed for detailed advances in our understanding of how small changes have ‘primed’ this algal lineage for multicellularity. However, the small-scale innovations observable in the genome cannot account for the variety of phenotypes that the clade itself possesses. For instance, there are similar abundances of transcription factors (TFs) between *Chlamydomonas* and *Gonium* (Hanschen et al., 2016), though there is evidence that certain TF families as well as other regulatory elements are contracting as developmental complexity increases in the multicellular volvocines (Jiménez-Marín et al., 2021). Making sense of these seemingly contradictory data is as challenging as it is promising, and some interesting insight is already becoming available.

The study of gene loss as a driver of evolutionary innovation is relatively recent (Go et al., 2005; Kvitek and Sherlock, 2013; Albalat and Canestro, 2016; Fernandez and Gabaldon, 2020; Guijarro-Clarke et al., 2020), but mathematical modeling suggests that gene loss might be related to changes to the molecular network of the impacted species in such a way that novel interactions might arise (Jiménez-Marín et al., 2021). This could translate to the ancestor of the colonial volvocines evolving multicellularity whilst setting up the resulting lineages for a wide array of developmental outcomes that might explain the discordance between the emergence of gene families, their co-option, and the emergence of more complex phenotypes among species.

**FIGURE 2:**
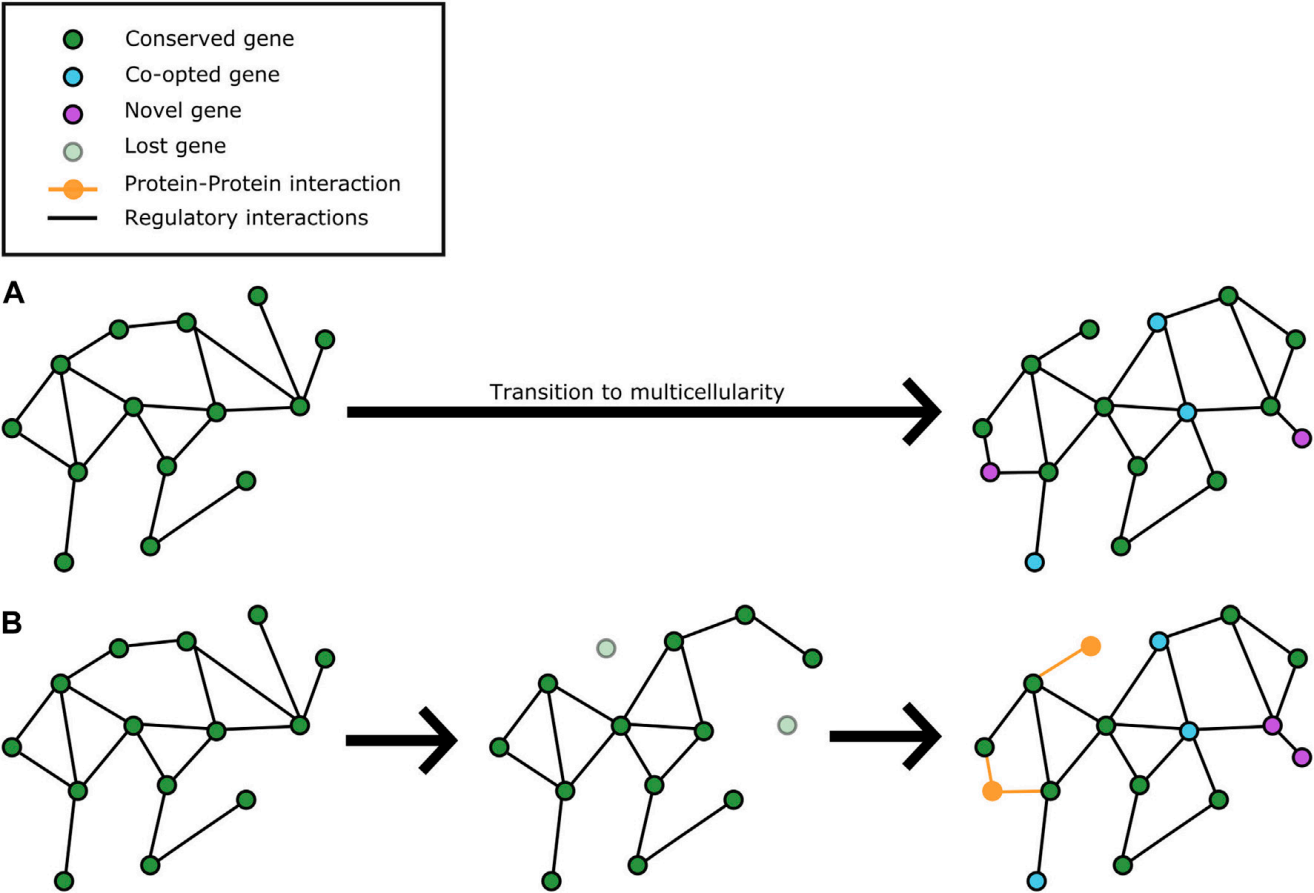
Gene loss contributes to molecular network rewiring for volvocine multicellularity in concert with limited co-option and expansion of functional units. **(A)** current understanding of how molecular networks in a unicellular, *Chlamydomonas*-like ancestor of the volvocines evolved upon the transition to multicellularity (Adami et al., 2000; Trigos et al., 2018). In this model, network elements are co-opted for multicellular function, and expanded to varying degrees for different multicellular lineages (magenta nodes) with minimal changes to the core “ancestral” (unicellular) network. **(B)** changes in molecular networks from a unicellular, *Chlamydomonas*-like ancestor of the volvocines by gene loss and limited co-option to evolve new functions for multicellularity. Some network elements were lost (light blue disconnected nodes), which caused a reconfiguration of the network to promote novel interactions (dotted magenta edges), co-option events (cyan nodes), and expansion of units with novel multicellularity related functions (magenta nodes).

Despite a general trend of reduction of histone copy numbers, *Gonium* histones H2B have a greater N-terminal variant diversity than other volvocines do (Jiménez-Marín et al., 2021). This might mean that differential combinations in histone tails between species could fuel differential post-translational change programs between species that could facilitate increases in developmental complexity even in the context of gene loss. Furthermore, protein-protein interaction (PPI) analysis of *Chlamydomonas, Gonium* and *Eudorina* strongly suggests that the proteomic makeup of each species is vastly different even for conserved gene products (Jiménez-Marín et al., 2021). These novel findings hint at the possibility that post-genomic programs have a strong role in generating and stabilizing more developmentally complex morphologies (Figure 2). Perhaps that is why it has proven so difficult to find “multicellularity genes”: differential usage of shared functional repertoires is a likely path to varying phenotypes that cannot be readily identified through comparative genomics alone.

### Combinatorial Co-Option and Creative Destruction

The elusive nature of the molecular players behind evolution of multicellularity and other Major Transitions in biological complexity might be due in part because they do not necessarily have specific genetic determinants. Rather, a few key master regulators of gene expression (e.g., *RB* and *regA*) undergo extensive co-option that results in a cascade of genome-wide developmental expression rewiring. In addition to this, it is feasible that epigenetic changes add to differential expression patterns between species; differences in histone diversity and abundance between species (Jiménez-Marín et al., 2021) provide some initial support to this possibility. Among the effects of global network rewiring is the formation of novel interactions at the protein level, which yields a combinatorial co-option of function that alters the organism’s functional repertoire without significantly shifting its genetic repertoire. In other words, rather than duplication and divergence to co-opt the function of genes, their combinations during development are altered for biological novelty. Functions that lose their adaptive value or become dispensable are gradually lost, as they are no longer needed.

The consequence of this framework is that, outside of a few key regulators, there may not be such a thing as “multicellularity genes”. Protein-protein interaction network rewiring should be capable of producing divergent phenotypes without the intervention of extensive genetic novelty. While on its face this mode of evolutionary novelty does not fit preconceptions, it is biologically sound. Unicellular ancestors were jack-of-all trades and had to perform many different functions. As they evolved into undifferentiated and differentiated multicellular organisms, these functions were either used as a “parts bin” for novel functions or their functions were no longer needed and lost. While increased sampling of volvocine genomes might increase the resolution on the history of key multicellularity genes, this framework hints at the need for the exploitation of other “omic” approaches (transcriptomics, proteomics) in conjunction with the usage and development of molecular biology tools (transformation, RNAi, CRISPR) that allow for comparisons between species and between WT and experimental strains under diverse conditions.

Upon transitioning to multicellularity, colonial organisms can switch from strictly temporal gene regulation to spatial or spatial temporal regulation patterns (Mikhailov et al., 2009). In the context that multicellularity has occurred numerous times under vastly different genetic backgrounds, it seems feasible that the many efforts to review the genetic underpinnings of this transition have come up short because multicellular evolution is not a process that requires a handful of genes to orchestrate organismal integration; rather, it requires a complete overhaul of how an organism uses whatever genes it has. Our future understanding of multicellularity will likely require a similar scientific overhaul, where the integration of information at different levels (DNA, RNA, phenotypic data) should lead to a more complete picture of the greater and lesser hurdles underlying this fascinating Major Transition.
